# A new climate data record of upper-tropospheric humidity from microwave observations

**DOI:** 10.1038/s41597-020-0560-1

**Published:** 2020-07-08

**Authors:** Theresa Lang, Stefan A. Buehler, Martin Burgdorf, Imke Hans, Viju O. John

**Affiliations:** 1 Universität Hamburg, Faculty of Mathematics, Informatics and Natural Sciences, Department of Earth Sciences, Meteorological Institute, Bundesstraße 55, 20146 Hamburg, Germany; 2 EUMETSAT, Eumetsat Allee 1, 64295 Darmstadt, Germany

**Keywords:** Atmospheric science, Climate change

## Abstract

We generated a new Climate Data Record (CDR) of Upper Tropospheric Humidity (UTH) based on observations from the microwave sounders Special Sensor Microwave Temperature - 2 (SSMT-2), Advanced Microwave Sounding Unit - B (AMSU-B) and Microwave Humidity Sounder (MHS). The data record covers the time period between 1994 and 2017 and provides monthly mean 183.31 ± 1 GHz brightness temperatures and derived UTH along with estimates of measurement uncertainty on a 1° × 1° latitude-longitude grid covering the tropical region (30° S to 30° N). For the UTH retrieval we introduce a new definition of UTH. Forgoing the use of the humidity Jacobian as a weighting function, it is easier to apply than the traditional definition without compromising the retrieval accuracy. The same definition can be used to derive UTH from infrared observations, allowing for a more synergistic use of infrared and microwave UTH in the future. The new UTH CDR is validated against an existing UTH data record.

## Abstract (summary)

**Table:** 

Measurement(s)	humidity
Technology Type(s)	Radiometry (Microwave)
Factor Type(s)	time period • geographic location
Sample Characteristic - Environment	troposphere • climate system

Machine-accessible metadata file describing the reported data: 10.6084/m9.figshare.12465830

## Background & Summary

In the framework of the Horizon 2020 project Fidelity and Uncertainty in Climate Data Records from Earth Observations (FIDUCEO) a new Level 3 Climate Data Record (CDR) of Upper Tropospheric Humidity (UTH) has been generated based on observations from the microwave humidity sounders Special Sensor Microwave Temperature - 2 (SSMT-2), the Advanced Microwave Sounding Unit - B (AMSU-B) and the Microwave Humidity Sounder (MHS). It covers the time period between 1994 and 2017 and provides monthly mean data on a 1° × 1° latitude-longitude grid covering the tropical region between 30° S and 30° N. UTH is derived from 183.31 ± 1 GHz brightness temperatures provided by the FIDUCEO Microwave Fundamental Climate Data Record (FCDR) Version 4.1^1,2^.

UTH is an important climate variable, because it has a significant impact on the Earth’s radiation budget^3^ and the associated water vapour feedback amplifies the climate system’s response to increases in other greenhouse gasses such as carbon dioxide^4^. Virtually all climate models show a water vapour feedback that is consistent with an approximately constant upper tropospheric relative humidity^5^. To evaluate climate model simulations, long-term observational data records of UTH are needed^6^.

Compared to existing UTH products, there are two major new aspects of the FIDUCEO UTH CDR: (1) A revised definition of UTH is used and (2) estimates of observational uncertainties are provided.

The first aspect is an attempt to solve a problem specifically related to the quantity UTH: Besides the microwave (MW) measurements used for this CDR, UTH can also be derived from observations in the infrared (IR) spectral region. However, comparing UTH derived from MW and IR measurements is hardly possible due to the traditional definition of UTH. UTH is traditionally defined as a weighted mean of the RH profile, where the weights are given by the humidity Jacobian. The humidity Jacobian peaks in the upper troposphere for both MW and IR water vapour channels. However, the exact shape of the Jacobian varies between channels, complicating a synergistic use of MW and IR UTH. We introduce a new UTH definition that is solely based on the vertical distribution of water vapour and forgoes the Jacobian. We show that this definition can be used to retrieve UTH from measurements by both microwave sounders (SSMT-2, AMSU-B and MHS) and the High-resolution Infrared Radiation Sounder 2 (HIRS/2) without loss in retrieval accuracy compared to the traditional approach. As HIRS/2 data is available in the time period from 1979 to 2016 it could be used at a later time to expand our UTH data record to earlier years. The new definition has the additional advantage that UTH can be calculated directly from given atmospheric profiles of humidity and temperature without a detour via radiative transfer simulations.

Detailed uncertainty information in CDRs derived from satellite-based Earth observations are needed to support the application of the data in climate research^7,8^. Providing such information on CDR level (level 2 or 3) has mainly been constrained by the availability of uncertainty information in the underlying FCDRs (level 1) in the past. Within the FIDUCEO project four new versions of such FCDRs were created, among them the FIDUCEO Microwave FCDR used as input for our UTH CDR. The FCDR includes information on observational uncertainty on pixel level, which is the result of rigorous uncertainty analyses based on metrological principles^9^. These uncertainties are propagated to the spatially and temporally averaged quantities in the UTH CDR. Depending on the spatial and temporal correlation behaviour of the underlying error sources, uncertainties are divided into three different classes, enabling the user to propagate them to spatial and temporal averages of the data.

The FIDUCEO UTH CDR is validated against an exisiting microwave UTH data record provided by the Satellite Application Facility on Climate Monitoring (CM-SAF). Differences in monthly tropical mean UTH do not exceed 2% RH and can be attributed to differences in the underlying FCDR and in the CDR processing in approximately equal parts.

The structure of this paper is as follows: The Methods chapter introduces the satellite instruments, the UTH retrieval method and the new definition of UTH. Furthermore, a detailed description of the CDR processing is provided. This is followed by the Data Records chapter, which includes a description of the CDR data file format as well as the satellite missions and time periods covered. The subsequent chapter Technical Validation consists of an evaluation of the UTH retrieval performance, the comparison of our CDR with the CM-SAF UTH CDR and a description of uncertainties not estimated in the CDR.

## Methods

### Instruments

The FIDUCEO UTH CDR combines measurements from the instruments Special Sensor Microwave Temperature - 2 (SSMT-2), the Advanced Microwave Sounding Unit - B (AMSU-B) and its successor, the Microwave Humidity Sounder (MHS). All three of them are passive microwave radiometers operating on polar-orbiting satellites. They have a similar design with two surface channels and three sounding channels located around the 183.31 GHz water vapour absorption line at 183.31 ± 1 GHz, 183.31 ± 3 GHz and 183.31 ± 7 GHz (183.31 + 7 for MHS), respectively. Typically the 183.31 ± 1 GHz channel is used to derive UTH since the signal reaching the instrument in this channel originates from the upper troposphere^10^. The channel has a total bandwidth of 1000 MHz for all three instruments. It is called channel H3 for MHS, channel 18 for AMSU-B and channel 2 for SSMT-2. For simplicity we will hereafter refer to the 183.31 ± 1 GHz channel of all instruments as the MW UTH channel.

Table 1 provides an overview of the scanning properties of MHS, AMSU-B and SSMT-2. All three are cross-track scanning instruments. For MHS^11^ each scan line consists of 90 Earth views, 45 on each side of the sub-satellite point. Each scan covers about 50° on both sides of the sub-satellite point, resulting in a swath width of 2180 km. The viewing angles range from 0.55° (with respect to the nadir view) to 48.95° in steps of 1.1°. With an antenna beamwidth of 1.1° the ground footprint at the innermost scan position has a diameter of approximately 16 km. The scanning geometry of AMSU-B^11^ is almost identical to that of MHS, only the viewing angles differ slightly from those of MHS. SSMT-2^12^ scans the Earth in only 28 views and has a larger beamwidth of 3.0°, resulting in a larger nadir footprint diameter of approximately 48 km.

**Table 1:** Basic scanning properties of SSMT-2, AMSU-B and MHS. Note that the numbers given for SSMT-2 are only valid for channel 2 since the beamwidth changes between the instrument channels.

Instrument	swath width [km]	nominal beam width [°]	nadir footprint diameter [km]	number of Earth views	innermost viewing angle [° from nadir]	outermost viewing angle [° from nadir]
SSMT-2	1400	3.0	48	28	1.5	40.5
AMSU-B	2250	1.1	16	90	0.55	48.95
MHS	2180	1.1	16	90	0.56	49.44

Numbers in this table are taken from^11,12^ and^15^.

**Table 2:** UTH scaling parameters determined from the ECMWF data set for all viewing angles of AMSU-B (a_*A*_, b_*A*_) and MHS (a_*M*_, b_*M*_).

Viewing angle	a_*A*_	b_*A*_	a_*M*_	b_*M*_
0.55	22.494	−0.09502	22.502	−0.09505
1.65	22.494	−0.09502	22.503	−0.09506
2.75	22.495	−0.09503	22.503	−0.09506
3.85	22.495	−0.09504	22.503	−0.09507
4.95	22.496	−0.09505	22.504	−0.09508
6.05	22.496	−0.09506	22.504	−0.09510
7.15	22.497	−0.09508	22.505	−0.09511
8.25	22.497	−0.09510	22.505	−0.09513
9.35	22.499	−0.09512	22.507	−0.09516
10.45	22.501	−0.09515	22.509	−0.09518
11.55	22.503	−0.09518	22.511	−0.09521
12.65	22.505	−0.09521	22.513	−0.09525
13.75	22.507	−0.09524	22.516	−0.09528
14.85	22.510	−0.09528	22.519	−0.09532
15.95	22.514	−0.09532	22.522	−0.09536
17.05	22.644	−0.09587	22.653	−0.09592
18.15	22.648	−0.09593	22.657	−0.09597
19.25	22.653	−0.09599	22.662	−0.09604
20.35	22.664	−0.09608	22.673	−0.09612
21.45	22.668	−0.09614	22.678	−0.09619
22.55	22.679	−0.09622	22.688	−0.09627
23.65	22.800	−0.09677	22.811	−0.09682
24.75	22.991	−0.09760	23.003	−0.09766
25.85	23.023	−0.09778	23.035	−0.09784
26.95	23.039	−0.09791	23.051	−0.09797
28.05	23.053	−0.09803	23.066	−0.09810
29.15	23.07	−0.09816	23.083	−0.09824
30.25	23.089	−0.09831	23.103	−0.09839
31.35	23.108	−0.09846	23.124	−0.09854
33.55	23.262	−0.09925	23.278	−0.09935
34.65	23.385	−0.09983	23.405	−0.09994
35.75	23.416	−0.10004	23.438	−0.10015
36.85	23.410	−0.10011	23.433	−0.10024
37.95	23.442	−0.10036	23.468	−0.10049
39.05	23.497	−0.10068	23.523	−0.10083
40.15	23.567	−0.10107	23.596	−0.10123
41.25	23.623	−0.10139	23.656	−0.10157
42.35	23.624	−0.10152	23.659	−0.10172
43.45	23.689	−0.10193	23.727	−0.10215
44.55	23.649	−0.10192	23.691	−0.10216
45.65	23.790	−0.10265	23.837	−0.10293
46.75	23.823	−0.10299	23.880	−0.10330
47.85	24.041	−0.10406	24.104	−0.10441
48.95	24.067	−0.10439	24.141	−0.10479

Viewing angles are given in degrees with respect to nadir and correspond to those of AMSU-B, which differ slightly from the exact viewing angles of MHS. The parameter *a* is dimensionless, *b* is in K^−1^.

**Table 3:** Satellite missions and time periods covered by the FIDUCEO UTH CDR.

Instrument	Satellite	Start	End
SSMT-2	DMSP F11	07/1994	04/1995
SSMT-2	DMSP F12	10/1994	01/2001
SSMT-2	DMSP F14	04/1997	01/2005
SSMT-2	DMSP F15	01/2000	01/2005
AMSU-B	NOAA15	01/1999	09/2010
AMSU-B	NOAA16	01/2001	05/2011
AMSU-B	NOAA17	10/2002	12/2009
MHS	NOAA18	08/2005	12/2017
MHS	NOAA19	11/2009	12/2017
MHS	MetopA	06/2007	12/2017
MHS	MetopB	01/2013	12/2017

For the development of a new UTH definition we simulated MW UTH channel brightness temperature (*T*_*b*_) with a radiative transfer model. Furthermore, since the aim is to find a common definition for MW and IR instruments, we additionally simulated *T*_*b*_ for an IR instrument, the High-Resolution Infrared Radiation Sounder (HIRS)^13^. HIRS is a cross-track scanning infrared radiometer with 20 frequency channels covering a spectral range from 0.69 to 15 μm. Channel 12 is designed to observe water vapour in the upper troposphere. For HIRS/2, the earliest version of the instrument, channel 12 is centred at 6.7 μm and has a spectral bandwidth of approximately 0.45 μm (2998 GHz). We will refer to it as the IR UTH channel in the following. When the instrument was upgraded to HIRS/3 with the launch of the satellite NOAA15 in 1998, the spectral response function of the channel changed and its centre wavelength moved to 6.5 μm^14^. Since only the 6.7 μm channel of the HIRS/2 instrument probes a similar altitude region of the atmosphere as the MW UTH channel, only the HIRS/2 instrument is considered here. HIRS scans through 56 Earth views, with instrument viewing angles ranging from 0.9° to 49.5° (from nadir), resulting in a total swath width of approximately 2240 km. With a field of view of 1.4° the footprint at the Earth’s surface has a size of 20.4 km at the innermost scan position^15^.

### UTH retrieval

To retrieve UTH from a measured brightness temperature *T*_*b*_ we make use of a linear relationship between the *T*_*b*_ and the logarithm of UTH, which was derived for 6.3 μm *T*_*b*_s^16^ and has also been successfully applied to microwave (183.31 ± 1 GHz) *T*_*b*_s^10^:

1 $${\rm{ln}}({\rm{UTH}})=a+b{T}_{b}.$$

**Fig. 1:**
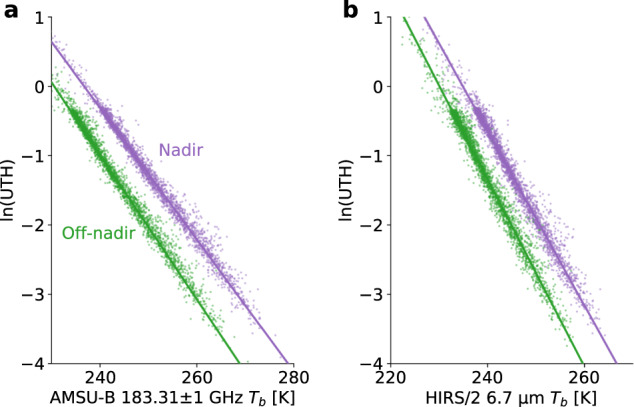
Linear regression to determine the UTH scaling parameters for two different satellite viewing angles. Logarithm of UTH versus AMSU-B 183.31 ± 1 GHz *T*_*b*_ (**a**) and HIRS/2 6.7 μm (**b**) for the nadir view (violet dots) and the most off-nadir view (green dots). Each dot corresponds to one training atmosphere in the 137-level ECMWF data set. The new UTH definition was used to calculate UTH for the training atmospheres. The linear fits to the data are indicated by solid lines.

The scaling parameters *a* and *b* are typically determined by a linear regression (Fig. 1) using a training data set of atmospheric temperature and humidity profiles. On the one hand, the *T*_*b*_ measured by the satellite instrument is simulated for every training profile using a radiative transfer model. On the other hand, UTH is calculated for each training atmosphere as an average of the vertical profile of relative humidity (RH) in a certain atmospheric layer in the upper-troposphere. The exact position of this layer depends on the chosen definition of UTH. A major requirement for a UTH definition is to fulfil Eq. (1). Note that all quantities in Eq. (1) depend on the viewing angle of the instrument.

**Fig. 2:**
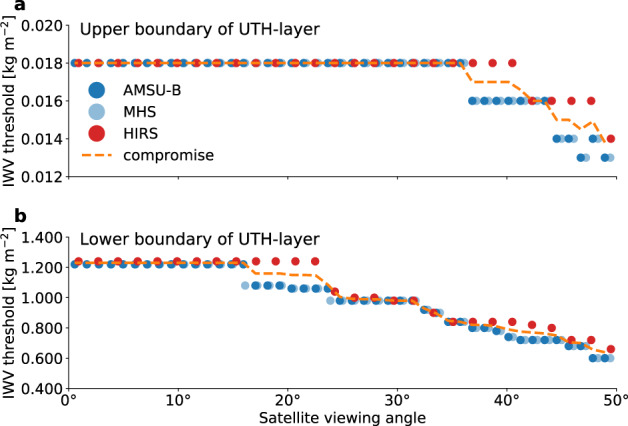
Optimised IWV thresholds for the new UTH definition. Most suitable IWV thresholds for the upper boundary (**a**) and lower boundary (**b**) of the UTH layer for AMSU-B (dark blue), MHS (light blue) and HIRS (red) at all instrument viewing angles (0° corresponds to the nadir view). For the final definition the mean of the thresholds determined for HIRS and the MW instruments (orange dashed line) is used.

**Fig. 3:**
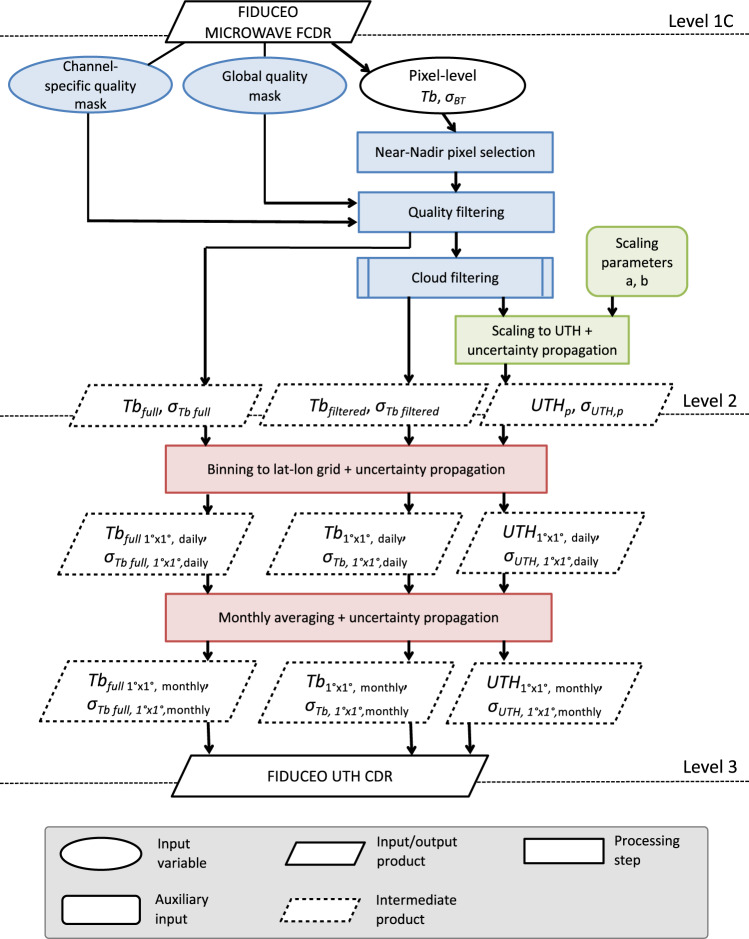
Schematic illustration of the FIDUCEO Microwave UTH CDR processing chain. The processing starts from the FIDUCEO Microwave FCDR and is subdivided into three main parts: pre-screening of pixels (blue), transformation of *T*_*b*_ to UTH (green) and gridding and temporal averaging (red). The processing chain consists of three main branches corresponding to the three core CDR variables *T*_*b*full_, *T*_*b*filtered_ and UTH.

**Fig. 4:**
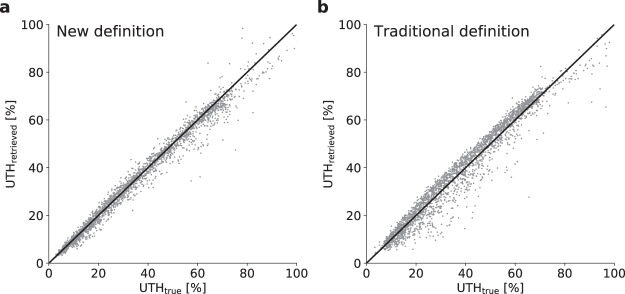
Performance of the new UTH retrieval compared to the traditional retrieval. UTH retrieved from AMSU-B nadir 183.31 ± 1 GHz brightness temperatures versus true UTH of all ECMWF training atmospheres for the retrieval with the new UTH definition (**a**) and the retrieval with the traditional UTH definition based on the VMR Jacobian (**b**).

**Fig. 5:**
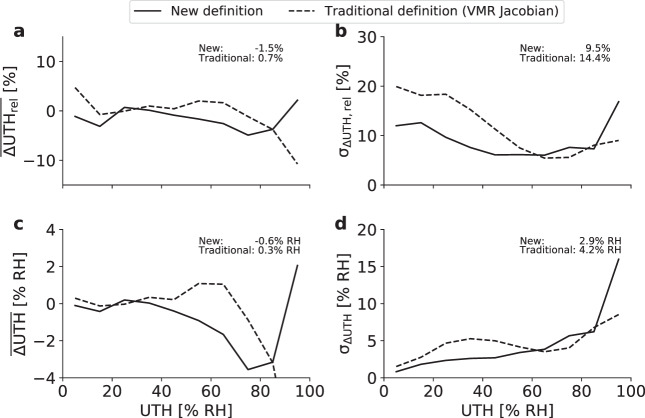
Retrieval statistics. Retrieval bias (**a**,**c**) and standard deviation (**b**,**d**) as a function of *UTH*_true_ in absolute units (**a**,**b**) and relative units (**c**,**d**) for the retrieval with the new definition (solid lines) and with the traditional definition based on the VMR Jacobian (dashed lines). Numbers in the upper right of each panel denote overall biases and standard deviations calculated from bins with UTH ≤ 80% RH.

**Fig. 6:**
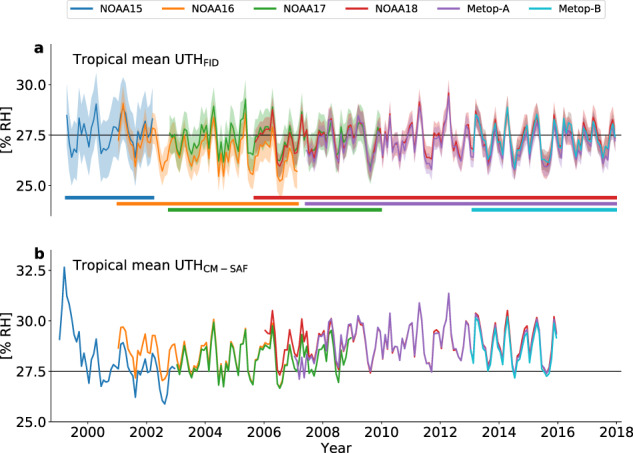
Time series of FIDUCEO UTH and CM-SAF UTH. Area-weighted monthly and tropical mean UTH from the FIDUCEO UTH CDR (**a**) and the CM-SAF UTH CDR (**b**) for all satellite missions contained in both CDRs. Black horizontal lines indicate a UTH of 27.5% RH. Shaded areas around the monthly means in (**a**) indicate the measurement uncertainty (±1 standard uncertainty), coloured horizontal bars indicate the time period covered by each satellite mission.

**Fig. 7:**
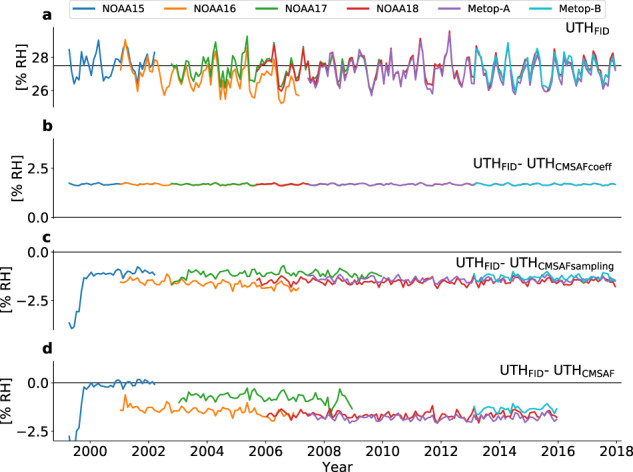
Difference of different CDR versions to the original FIDUCEO UTH CDR. Area-weighted monthly and tropical mean UTH from the FIDUCEO UTH CDR (**a**, same as in Fig. 6) and differences between UTH from the original FIDUCEO UTH CDR and the version generated with the scaling coefficients of the CM-SAF UTH CDR (**b**), the version generated using all pixels (**c**) and the CM-SAF UTH CDR (**d**). Black horizontal lines indicate a UTH of 27.5% RH in (**a**) and zero difference in (**b**–**d**), respectively.

**Fig. 8:**
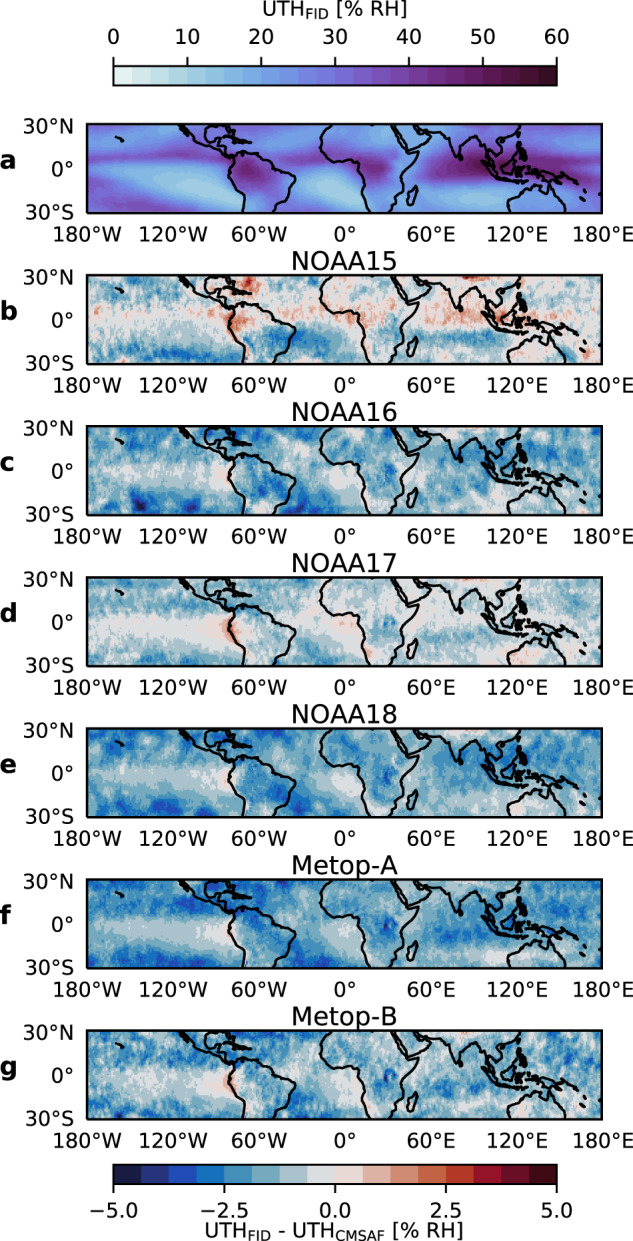
Geographical distribution of mean differences UTH_FID_ – UTH_CM–SAF_. Mean distribution of UTH_FID_ in the tropics from the complete time series of NOAA18 (**a**) and distribution of mean differences UTH_FID_ – UTH_CM–SAF_ for all satellite missions contained in both CDRs: NOAA15 (**b**), NOAA16 (**c**), NOAA17 (**d**), NOAA18 (**e**), Metop-A (**f**) and Metop-B (**g**).

**Fig. 9:**
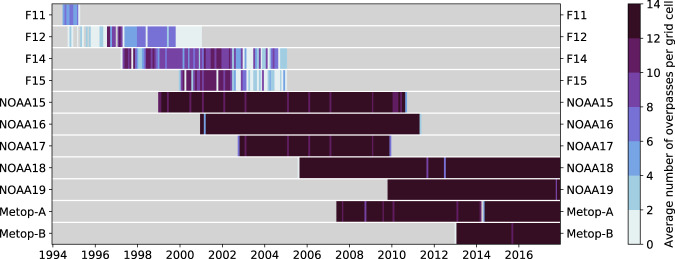
Coverage of the different satellites included in the FIDUCEO UTH CDR. Average number of satellite overpasses per grid cell and month (colour shading) for each satellite mission. Grey areas indicate time periods for which no data is available.

**Fig. 10:**
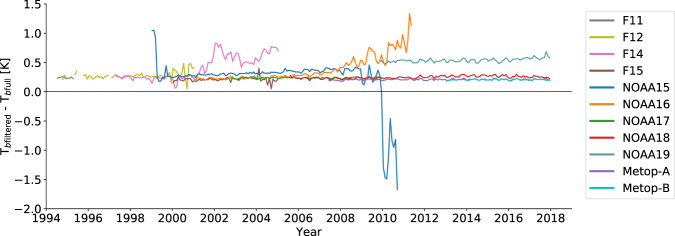
Impact of cloud filtering on *T*_*b*_ in the FIDUCEO UTH CDR. Difference between area-weighted tropical monthly means of *T*_*b*full_ and *T*_*b*filtered_ for all satellite missions (colors). The black line indicates zero difference. For most of the satellite missions the difference is about 0.2 K, but for F14, NOAA15, NOAA16 and NOAA19 the cloud filtering has a stronger impact on *T*_*b*_.

Traditionally, UTH is defined as a weighted mean of the RH profile, where the weights are given by the humidity Jacobian for the respective instrument channel. Relation 1 is fulfilled with this traditional definition because the altitude levels weighted strongest by the humidity Jacobian correspond to the atmospheric emission layer, i.e. the atmospheric layer contributing to the measured *T*_*b*_. This Jacobian-based definition has the disadvantage that radiative transfer simulations have to be performed whenever UTH needs to be calculated for given atmospheric profiles of temperature and humidity. Moreover, even though the UTH channels of MW and IR instruments are sensitive to RH in a very similar altitude range, the exact shape of the corresponding humidity Jacobians are different. The resulting difference in the definitions of IR and MW UTH complicate a synergistic use. Therefore, we adapt the traditional UTH definition for the FIDUCEO UTH CDR by eliminating the humidity Jacobian. This new UTH definition will be explained in the next section. The setup used to determine new scaling parameters *a* and *b* is described in the following.

Our training data consist of atmospheric profiles from the 137-level sampled ECMWF data set^17^, which is compiled from the short-range forcast by the Integrated Forecasting System (IFS) spanning the time period of 1 September 2013 to 31 August 2014. The complete data set consists of five subsets with 5000 profiles each, corresponding to the sampling for a specific geophysical variable. For the UTH regression we choose the subset that focuses on a diverse sampling of atmospheric humidity. For this subset 10% of profiles were selected in such a way that differences in specific humidity between the sampled profiles are maximized. The remaining 90% of profiles were selected randomly to include a realistic amount of frequently occurring atmospheric states. Despite this large proportion of randomly sampled profiles extreme humidity cases are overrepresented in the sampled distribution due to the diverse sampling of humidity. However, since we use the profiles to perform a linear regression, a high variability in humidity is as important as a realistic frequency distribution. Therefore, the dataset is a suitable choice for this application. Since the UTH CDR will be restricted to the tropical region we select all tropical profiles from the 5000 profiles in the subset. This leaves a set of 2812 tropical training profiles.

A line-by-line radiative transfer model, The Atmospheric Radiative Transfer Simulator (ARTS) Version 2.2^18,19^, is used to simulate clear-sky 183.31 ± 1 GHz *T*_*b*_ for all viewing angles of AMSU-B and MHS. As our new definition is supposed to work also for IR UTH retrievals, we additionally simulate 6.7 μm *T*_*b*_. Required geophysical inputs for the radiative transfer simulations are atmospheric profiles of humidity, temperature, ozone, oxygen and nitrogen as well as the surface skin temperature and the surface emissivity. Humidity, temperature and ozone profiles are taken from the training data set. Volume mixing ratios of nitrogen and oxygen are assumed to be constant throughout the atmosphere. Spectroscopic parameters are taken from the high-resolution transmission molecular absorption database (HITRAN) catalogue^20^. The skin temperature was assumed to be equal to the lowest atmospheric temperature and surface emissivities of 0.6 and 1 were used for the simulation of the MW instruments and HIRS/2, respectively. Due to the strong absorption of water vapour in the UTH channels the surface emissivity only influences the simulated *T*_*b*_ under extremely dry conditions or above high mountains. These extreme cases are filtered out as explained in the next paragraph. To remove such cases conservatively, we assume a rather low value of 0.6 for the MW surface emissivity, which roughly corresponds to the emissivity of an ocean surface.

Surface-contaminated cases are removed using the same methodology as^10^: Profiles are discarded if the 183.31 ± 7 GHz *T*_*b*_ of AMSU-B is not higher than the 183.31 ± 1 GHz *T*_*b*_. Under clear-sky conditions this only occurs when both channels see the surface. For all other cases, the 183.31 ± 7 GHz *T*_*b*_ is higher, because the emission originates from lower tropospheric levels. Due to the similarity of the HIRS and AMSU-B UTH channels, it is assumed that surface contamination in the HIRS channel occurs for the same profiles than in the AMSU-B channel. As expected surface contamination is rare for our tropical training profiles. From 2812 profiles only 12 are filtered out by our algorithm.

The remaining profiles are used to determine the scaling parameters *a* and *b* from the linear regression of ln(UTH) against *T*_*b*_. The regression is shown in Fig. 1 for the nadir view and the most off-nadir view of AMSU-B and HIRS. The linear relation (Eq. (1)) is well fulfilled for both viewing angles, indicating that the retrieval error is small. The retrieval performance is assessed in more detail in the validation section of this paper. Regression coefficients for all viewing angles of AMSU-B and MHS are listed in Table 2.

### New definition of UTH

The new definition is based on the concept that the atmospheric emission layer for a water vapour channel is bounded by two characteristic amounts of water vapour integrated from the top of the atmosphere downwards^21^. Using this idea, we define UTH as the mean RH in a layer between two altitude levels *z*(*IWV*_1_) and *z*(*IWV*_2_), at which the integrated water vapour (*IWV*) above exceeds two viewing angle dependent thresholds *IWV*_1_ and *IWV*_2_:

2 $${{\rm{UTH}}}_{{\rm{new}}}(\theta )=\frac{1}{z(IW{V}_{1}(\theta ))-z(IW{V}_{2}(\theta ))}\underset{z(IW{V}_{2}(\theta ))}{\overset{z(IW{V}_{1}(\theta ))}{\int }}RH(z)dz,$$

where *θ* is the satellite viewing angle and RH is defined relative to liquid water. The thresholds *IWV*_1_ and *IWV*_2_ play a similar role in capturing the atmospheric emission layer as the Jacobian in the traditional definition. Since the emission layer is similar for the MW instruments and the HIRS/2 instrument, it is possible to use the same IWV thresholds and hence the same UTH definition for both instrument types.

The IWV thresholds were optimised in such a way that the linear relationship between the *T*_*b*_ and the logarithm of UTH (Eq. (1)) is best fulfilled for the ECMWF training atmospheres. For the optimisation the above-described linear regression was repeatedly performed with different combinations of IWV thresholds in the UTH definition. For each instrument viewing angle the pair of thresholds used for the regression with the smallest root mean square error was chosen to be the most suitable one.

The optimised IWV thresholds are visualized in Fig. 2 for AMSU-B, MHS and HIRS/2. Both thresholds *IWV*_1_ and *IWV*_2_ exhibit a dependence on the instrument viewing angle. The thresholds decrease as the viewing direction of the satellite moves away from nadir because the path length through the atmosphere increases and a given IWV along the sensor’s line of sight is reached in higher altitudes. Since the IWV above is always defined along the vertical direction, a higher altitude corresponds to a smaller IWV above. Note that the viewing angle dependence of the IWV thresholds implies that the definition of UTH depends on the satellite viewing angle.

The optimal thresholds for the MW instruments AMSU-B and MHS are identical for nearly all viewing angles. This is not surprising since the UTH channels of the two instruments are designed almost identically. For SSMT-2 the optimisation was not performed separately, but it is assumed that the optimal thresholds are similar to those of AMSU-B and MHS due to the similar instrumental design.

The IWV thresholds for HIRS/2 have similar magnitudes and show the same dependence on the satellite viewing angle as the thresholds for the MW instruments. Therefore, MW and HIRS/2 thresholds are averaged to obtain compromise thresholds, indicated by the dashed line in Fig. 2. The use of these compromise thresholds ensures that the final UTH definition is identical for both the MW sensors and HIRS/2. We will show in the validation section that the performance of the UTH retrieval does not suffer from the new UTH definition, confirming that the use of compromise thresholds is reasonable.

### CDR processing

The new UTH scaling parameters *a* and *b* were applied in the creation of a new level 3 UTH CDR based on 183.31 ± 1 GHz *T*_*b*_ from the level 1c FIDUCEO Microwave FCDR. The three core variables contained in the FIDUCEO UTH CDR are monthly mean 183.31 ± 1 GHz *T*_*b*_ derived from all pixels (*T*_*b*full_) and from cloud-filtered pixels (*T*_*b*filtered_), as well as UTH derived from cloud-filtered *T*_*b*_. The variables are mapped on a regular 1° × 1° latitude-longitude grid covering the tropical region. The CDR also provides measurement uncertainties for all quantities. They are propagated from the underlying FCDR.

The processing chain (Fig. 3) consists of three main branches corresponding to the three core CDR variables *T*_*b*full_, *T*_*b*filtered_ and UTH. In the following, we provide a more detailed description of the input data for the CDR and the CDR processing including the propagation of uncertainty. For this description we concentrate on the branch corresponding to the core variable UTH since it encompasses all important processing steps. These comprise a pre-screening of pixels, the transformation from *T*_*b*_ to UTH as well as spatial and temporal averaging.

#### Input data: The FIDUCEO Microwave FCDR

The input data for the UTH CDR is provided by the FIDUCEO Microwave FCDR Version 4.1^1,2^. It contains *T*_*b*_s from all instrument channels along with measurement uncertainties for 11 satellite missions carrying either SSMT-2, AMSU-B or MHS between 1994 and 2017. As for all level 1 products the data are provided on pixel level, i.e. the dimensions of the data correspond to the directions along and across the satellite ground track.

Novelties of the FIDUCEO Microwave FCDR include the use of a metrology inspired measurement-equation approach^9^ for the re-calibration of brightness temperatures and the estimation of measurement uncertainty. For the letter all influences (effects) on the instrumental calibration that lead to errors in the observed signal are taken into account and the associated uncertainties are estimated and propagated to *T*_*b*_ through the measurement equation. See^1^ for a more detailed description of this procedure. Measurement uncertainty is split into three classes, depending on the type of the underlying effect and the resulting error correlation:

- Uncertainties due to independent effects (hereafter independent uncertainties): The underlying effects are close to white noise and generate a completely independent uncertainty from pixel to pixel.
- Uncertainties due to structured effects (hereafter structured uncertainties): Even though the underlying effects are random, the calibration procedure can lead to correlations between pixels that are located close to each other. The resulting correlations have spatial and temporal scales that are typically smaller than one satellite orbit.
- Uncertainties due to common effects (hereafter common uncertainties): This class includes all effects with associated correlation scales larger than one orbit (often the whole satellite mission).

#### Pre-screeing of pixels

In a pre-screening procedure, a subset of pixels is selected from the pixel-level 183.31 ± 1 GHz brightness temperatures for further processing.

First, only pixels located close to the nadir view of the satellite are selected. As pointed out earlier, the UTH definition depends on the satellite viewing angle because the UTH layer shifts to higher altitudes as the viewing direction of the satellite moves away from nadir. Since we do not want to mix UTH values defined in different ways, we select only pixels characterized by uniform IWV thresholds (Fig. 2) and hence a uniform UTH definition. This comes at the expense of spatial and temporal coverage but simplifies comparisons with UTH calculated directly from atmospheric profiles, e.g. from model output or radiosonde measurements. For AMSU-B and MHS IWV thresholds are constant for the innermost 28 pixels of the scan line (14 on both sides of the nadir views). This corresponds to the innermost 10 pixels of SSMT-2, assuming that the IWV thresholds for SSMT-2 are similar to those of AMSU-B and MHS.

Second, pixels of low quality are discarded. The FCDR provides quality information for each pixel in the form of an overall quality bitmask and a channel-specific bitmask. Pixels that are marked as “invalid” by the overall quality bitmask are rejected. This can have several reasons, e.g. invalid geolocation or viewing-geometry of the data, invalid pixel acquisition time or invalid sensor status. For a full list of reasons see the description of the quality bitmasks in^22^. Pixels are also discarded if the specific bitmask of the 183.31 ± 1 GHz channel indicates that calibration was not possible or there was bad data from the Earth views.

Third, cloud contaminated pixels are removed. UTH can only be derived from measurements of *T*_*b*_ that are not contaminated by clouds. A strong advantage of MW measurements over IR measurements is the fact that clouds are nearly transparent in the MW. However, cold ice clouds do interact with MW radiation and can affect the measurement^23^. The cloud particles scatter radiation away from the sensor’s line of sight and hence cause a reduction in *T*_*b*_. A cloud filtering has to be performed before retrieving UTH in order to avoid a positive UTH bias in the climatology due to the erroneous interpretation of cloudy scenes as very moist scenes^24^. This is done using the method suggested in^25^, which combines two criteria. The first criterion is a viewing angle dependent threshold on the 183.31 ± 1 GHz *T*_*b*_ (240.1 K for the Nadir view). This threshold is based on simulated clear-sky *T*_*b*_, which were shown to lie above this value. The second criterion uses the differences between 183.31 ± 1 GHz *T*_*b*_ and 183.31 ± 3 GHz *T*_*b*_. Under clear-sky conditions, the 183.31 ± 1 GHz *T*_*b*_ is colder than the 183.31 ± 3 GHz *T*_*b*_, because the former is sensitive to a higher region in the troposphere, where temperatures are generally lower. However, in the presence of ice clouds the 183.31 ± 1 GHz *T*_*b*_ can be warmer than the 183.31 ± 3 GHz *T*_*b*_. Hence, the difference between the two *T*_*b*_ can be used to detect clouds. Additionally, as shown by^25^, this difference is also a good filter against surface influence in the 183.31 ± 1 GHz *T*_*b*_.

#### Transformation of brightness temperature to UTH

After the pre-screening UTH is calculated for each pixel *p* from cloud-filtered *T*_*b*_ (*T*_*b*,filtered_) using Eq. (1) and the scaling parameters *a* and *b* derived in the UTH retrieval section:

3 $$UT{H}_{p}=exp(a+b{T}_{b,{\rm{filtered}}}),$$

For SSMT-2 the scaling coefficients were not derived separately, but the coefficients of the respective nearest MHS views are used. This is a reasonable simplification since the UTH channels of SSMT-2 and MHS have very similar characteristics.

#### Pixel aggregation and averaging

After the calculation of pixel-level *UTH*_*p*_, data from different pixels are combined in a two-step process. In the first step all pixels from observations of one day are aggregated in 1° × 1° bins on a regular latitude-longitude grid covering the tropics. Subsequently, the aggregated pixels are averaged to get daily averages of *UTH* for each day *d* and each grid cell (*UTH*_1°×1°,*d*_):

4 $$UT{H}_{{1}^{\circ }\times {1}^{\circ },d}=\frac{1}{N}\mathop{\sum }\limits_{p=1}^{N}UT{H}_{p}.$$

Here, *N* is the number of pixels that are aggregated within the 1° × 1° region. In a second step, daily averages are combined to monthly means for every grid cell:

5 $$UT{H}_{{1}^{\circ }\times {1}^{\circ },m}=\frac{1}{{N}_{d}}\mathop{\sum }\limits_{d=1}^{{N}_{d}}UT{H}_{{1}^{\circ }\times {1}^{\circ },d},$$

where the index *m* denotes the month and *N*_*d*_ is the number of daily averages entering the monthly average.

#### Propagation of uncertainty

The uncertainties of pixel-level brightness temperatures from the FCDR are propagated to the gridded and averaged *UTH* values in the CDR using the Law of the Propagation of Uncertainties (LPU)^26^, which yields the uncertainty *u* of a quantity *y*, which is determined from *m* other quantities *x*_1_, *x*_2_, …, *x*_*m*_ through a functional relationship *f*:

6 $$u(y)=\sqrt{\mathop{\sum }\limits_{i=1}^{m}{\left(\frac{\partial f}{\partial {x}_{i}}\right)}^{2}u{({x}_{i})}^{2}+2\mathop{\sum }\limits_{i=1}^{m-1}\mathop{\sum }\limits_{j=i+1}^{m}\frac{\partial f}{\partial {x}_{i}}\frac{\partial f}{\partial {x}_{j}}u({x}_{i})u({x}_{j})r({x}_{i},{x}_{j})},$$

where *u*(*x*_*i*_) are the uncertainties of the input quantities *x*_*i*_. The partial derivatives of *f* with respect to the input quantities *x*_*i*_ describe the sensitivity of *f* to changes in *x*_*i*_ and are therefore also called sensitivity coefficients. The correlation coefficient *r*(*x*_*i*_, *x*_*j*_) characterizes the correlation between *x*_*i*_ and *x*_*j*_. Hence, positive correlations between the input quantities *x* increase the uncertainty of the output quantity *y*. To account for the different error correlation properties of independent, structured and common uncertainties, each class of uncertainty is propagated separately.

In a first step, uncertainties of pixel-level *T*_*b*,filtered_ are propagated to pixel-level *UTH*_*p*_. This unit transformation (Eq. (3)) has only one input quantity *T*_*b*,filtered_ and the LPU (Eq. (6)) reduces to:

7 $${u}_{c}(UT{H}_{p})=\sqrt{{\left(\frac{\partial UT{H}_{p}}{\partial {T}_{b,{\rm{filtered}}}}\right)}^{2}{u}_{c}{\left({T}_{b,{\rm{filtered}}}\right)}^{2}}=\left|bUT{H}_{p}{u}_{c}({T}_{b,{\rm{filtered}}})\right|,$$

where the index *c* denotes the class of uncertainty.

Subsequently, uncertainties are propagated from pixel-level *UTH* to daily grid cell averages *UTH*_1°×1°,*d*_ (Eq. (4)). For this averaging process, the LPU (Eq. (6)) takes the following form:

8 $${u}_{c}(UT{H}_{{1}^{\circ }\times {1}^{\circ },d})=\frac{1}{N}\sqrt{\mathop{\sum }\limits_{p=1}^{N}{u}_{c}{(UT{H}_{p})}^{2}+2\mathop{\sum }\limits_{p=1}^{N-1}\mathop{\sum }\limits_{p{\prime} =p+1}^{N}{u}_{c}(UT{H}_{p}){u}_{c}(UT{H}_{p{\prime} })r(p,p{\prime} )},$$

where *r*(*p*, *p*′) is the correlation coefficient of two pixels denoted by *p* and *p*′. For independent uncertainties the correlation is zero, so the second term under the square root vanishes. Consequently, in this case the averaging process significantly reduces the uncertainty of the grid cell value compared to the uncertainties of the individual pixel values. If all *N* pixel uncertainties were equal, the grid cell uncertainty would be reduced by a factor 1/$\sqrt{N}$. The opposite is true for the common uncertainties. For this class correlations may extend over infinite length and time scales and the correlation coefficient *r* is one per definition. Thus, averaging of several pixel values does not reduce the grid cell uncertainty. In other words, the grid cell uncertainty is obtained by averaging the pixel uncertainties. For structured uncertainties, correlations extend over a certain number of adjacent scan lines in the satellite swath. This results from an averaging of calibration coefficients over several scan lines. In the case of the Microwave FCDR, calibration coefficients are always averaged over seven scan lines^22^, so the overall length scale over which the correlation decreases to zero is seven scan lines. The FCDR contains so-called correlation vectors *ρ*, providing the correlation coefficient of two pixels *p* and *p*′ as a function of the difference between the scan lines *l* of these pixels |*l*_*p*_ − *l*_*p*′_|. Using these correlation vectors *ρ* the correlation coefficient *r* in Eq. (8) can be written as

9 $$r(p,p{\prime} )=\rho \left(\left|{l}_{p}-{l}_{p{\prime} }\right|\right).$$

The uncertainty propagation from daily averages *UTH*_1°×1°,*d*_ to the monthly average *UTH*_1°×1°,*m*_ (Eq. (5)) is performed in a very similar way, under the assumption that there is no temporal correlation (*r* = 0) for independent and structured uncertainties and full temporal correlation (*r* = 1) for common uncertainties.

Error correlations between the pixel-level input quantities also result in correlations between the final grid cell averages. These “inter-grid cell correlations” are not included in the CDR. However, they must be taken into account in further spatial or temporal averaging and the associated uncertainty propagation should be performed by the CDR user. For independent and common uncertainties the correlations among grid cells behave in the same way as those among pixels; for independent uncertainties there are no correlations, whereas for common uncertainties all grid cells are fully correlated. For structured uncertainties, the correlation structure is more complex and a complete propagation that also yields the covariances of the grid cell averages has to be performed. For one thing, however, this requires large covariance matrices and hence much computational power, and for another, it is a complex procedure to take into account non-uniform inter-grid cell correlation patterns for further propagation of uncertainties. Therefore, we recommend to treat the structured uncertainties in the same way as common uncertainties in an averaging process. This approach provides an upper limit for the structured uncertainty of the average.

## Data Records

The FIDUCEO Microwave UTH CDR version 1.2^27^ is freely available from the Centre for Environmental Data Analysis (CEDA) Archive.

### Description of data files

The CDR data files are written in NetCDF-4 format, implementing the common CDR file format that has been defined within the FIDUCEO project. Each file contains data for one month and one satellite mission and has a size of about 3.9 MB, resulting in a total CDR size of about 4.0 GB. The filenames follow the FIDUCEO standard and have the following structure:

FIDUCEO_CDR_UTH_{INSTRUMENT}_{SATELLITE}

_{STARTTIME}_{ENDTIME}_L3_v{CDR-VERSION}

_fv{WRITER-VERSION}.nc

where {INSTRUMENT} can be either SSMT2, AMSUB or MHS. {SATELLITE} can be any of the satellites these instruments are flying on. {STARTTIME} is the first second of the first day in the month, with the format year-month-day-hour-minute-second, {ENDTIME} is the last second of the last day in the month. {CDR-VERSION} denotes the version number of the CDR and {WRITER-VERSION} is the version of the NetCDF-writer used to generate the NetCDF files.

Each data file consists of global attributes with general information and a set of data variables with individual attributes. The global attributes provide information on the version of the CDR and the time period covered by the data file. Moreover, they contain the Digital Object Identifier (DOI) of the data set. In order to sustain traceability, a list of file names of all FCDR files that were used to generate the CDR file is also included in the global attributes.

The following variables are contained in each file:

- lon, lat: Longitudes and latitudes of grid cell centres;
- BT_full, BT_full_inhomogeneity: Monthly average and standard deviation of 183.31 ± 1 GHz *T*_*b*_ from all available pixels (including cloudy pixels);
- BT, BT_inhomogeneity: Monthly average and standard deviation of 183.31 ± 1 GHz *T*_*b*_ from all pixels used to derive UTH (excluding cloudy pixels);
- uth, uth_inhomogeneity: Monthly average and standard deviation of UTH;
- u_independent, u_structured, u_common: Independent, structured and common uncertainty for monthly averages of *T*_*b*_ and UTH (denoted by suffixes _BT and _uth, respectively);
- observation_count: Number of pixels entering the monthly grid cell average;
- overpass_count: Number of satellite overpasses contributing to the monthly grid cell average;
- time_ranges Earliest and latest time of day of pixel contribution to the monthly average;
  All monthly fields are split into two parts: One from ascending satellite overpasses and one from descending overpasses, indicated by the suffixes _ascending and _descending, respectively, in the variable names. For each variable several attributes are provided. They contain a description of the quantity as well as information on the unit and the dimensions of the variable. Existing dimensions are:
- x - East-west dimension (size: 360);
- y - North-south dimension (size: 61);
- bounds - Dimension defining lower and upper bounds (size: 2);

Another attribute is the fill value, which is placed whenever there are data gaps due to missing FCDR data or due to cloud coverage.

### Covered satellite missions and time periods

Similar to the underlying Microwave FCDR, the FIDUCEO UTH CDR covers the time period from 1994 to 2017 and consists of 11 partly overlapping satellite missions carrying either SSMT-2, AMSU-B or MHS. An overview of all included satellite missions and the corresponding time periods is provided in Table 3.

## Technical Validation

### Performance of the UTH retrieval

To evaluate the performance of the UTH retrieval with the new UTH definition (new retrieval) it is compared to the performance of a retrieval with the traditional definition based on the fractional water vapour mixing ratio Jacobian as in^10^ (traditional retrieval). We determined the UTH scaling parameters *a* and *b* for the traditional retrieval based on the same ECMWF training data set we used for the new retrieval.

To give an impression of the retrieval performance Fig. 4 shows the retrieved UTH (*UTH*_retrieved_) from AMSU-B *T*_*b*_ versus the true UTH (*UTH*_true_) for all ECMWF training atmospheres for both retrievals. The scatter of the data points around the one to one line seems to be approximately equal for both retrievals, indicating that the retrieval accuracy does not suffer from the new definition.

To assess the retrieval performance in more detail, the difference Δ*UTH* between *UTH*_retrieved_ and *UTH*_true_ is calculated for all training atmospheres:

10 $$\Delta UTH=UT{H}_{{\rm{retrieved}}}-UT{H}_{{\rm{true}}}.$$

Following^10^ we define the retrieval bias as the mean of Δ*UTH* of all training atmospheres ($\bar{\Delta UTH}$) and the retrieval standard deviation as the standard deviation of Δ*UTH* (*σ*_Δ*UTH*_). Relative retrieval bias ($\bar{\Delta UT{H}_{{\rm{r}}{\rm{e}}{\rm{l}}}}$) and relative retrieval standard deviation (${\sigma }_{\Delta UT{H}_{{\rm{rel}}}}$) are defined similarly, but based on the relative difference between *UTH*_fitted_ and *UTH*_true_:

11 $$\Delta UT{H}_{rel}=\frac{UT{H}_{{\rm{retrieved}}}-UT{H}_{{\rm{true}}}}{UT{H}_{{\rm{true}}}}.$$

We calculate retrieval statistics by aggregating the data points in 10% RH bins of *UTH*_true_ values. Absolute and relative retrieval bias and standard deviation as a function of *UTH*_true_ are shown in Fig. 5. Overall retrieval bias and standard deviation were calculated only from profiles with UTH ≤ 80% RH since higher values of RH with respect to water hardly occur in nature^28^ and seem to be a particularity of the ECMWF data set.

Using the new definition, the absolute retrieval bias fluctuates between −3% and 1% for UTH values below 40% RH and increases in magnitude to about −3.5% for UTH values of 70–80% RH. The relative bias is between 0.1% and −4% in all UTH bins below 80% RH. These biases are slightly larger than for the retrieval with the traditional definition for UTH values above 40% RH and slightly smaller for UTH values below 40% RH. With the new definition the absolute retrieval standard deviation in the 0–10% UTH bin is approximately 1% RH and continuously increases to approximately 6% RH at 70–80% UTH. In relative units, the retrieval standard deviation decreases from about 12–13% at UTH values below 20% RH to about 6–8% for UTH values above 40% RH. Compared to the retrieval with the traditional definition, standard deviations are generally lower for UTH below 60% RH and slightly higher for UTH above 60% RH.

For the new retrieval the overall absolute (relative) retrieval standard deviation is 2.9% RH (9.5%), for the traditional retrieval it is 4.2% RH (14.4%). The overall absolute (relative) retrieval bias is −0.6% RH (−1.5%) for the new retrieval and 0.3% RH (0.7%) for the traditional one. Hence, in terms of overall standard deviation the performance of the new retrieval has slightly improved with respect to the traditional one, in terms of overall retrieval bias it has slightly worsened. It is important to note here that the newest version of the CM-SAF UTH CDR, which we will use for comparison in the next section, uses an updated UTH definition based on the RH Jacobian instead of the VMR Jacobian. With the RH Jacobian the retrieval standard deviation is reduced compared to the VMR Jacobian and is similar as with the new definition^29^. We conclude that the retrieval performance does not suffer from our new UTH definition compared to the two traditional versions used in previous datasets.

### Comparison to the CM-SAF UTH CDR

To validate the FIDUCEO UTH CDR, it is compared to the Microwave UTH data record provided by the European Organisation for the Exploitation of Meteorological Satellites (EUMETSAT) Satellite Application Facility on Climate Monitoring (CM-SAF)^30^, hereafter called CM-SAF CDR. We chose the comparison to an existing UTH data record over a comparison with in-situ measurements from radiosondes for two main reasons. Firstly, humidity measurements from many types of radiosondes are subject to significant biases in the upper troposphere^31,32^ and these biases strongly depend on the sensor type^33^. Hence, combining different sensor types, which would be required to get a sufficient temporal and spatial coverage for the validation of a UTH CDR, is problematic. Even if the quality of the radiosonde data is good, direct comparisons of satellite and radiosonde measurements of water vapour remain difficult due to their different nature and different measurement scales^34^, which has lead to a large spread in bias results^35^. Secondly, it is of particular interest how the new aspects of our CDR with respect to existing data records are reflected in UTH. Our CDR differs from the CM-SAF CDR in the underlying FCDR and several aspects of processing, including the UTH scaling parameters (due to a different UTH definition) and the pixel selection.

The CM-SAF UTH CDR is based on a microwave humidity sounder FCDR generated by EUMETSAT within the framework of the European Reanalysis of Global Climate Observations 2 (ERA-Clim2) project. The approach used to reduce inter-satellite biases in this FCDR is a bias-correction based on “Observation minus Background” (O-B) statistics from the ERA-Interim reanalysis and hence differs from the measurement equation based recalibration approach used for the FIDUCEO FCDR. Thus, it can be expected that inter-satellite biases differ between our new CDR and the CM-SAF CDR. To determine the scaling coefficients for the UTH retrieval in the CM-SAF CDR a similar linear regression approach as in the FIDUCEO CDR was used, but UTH was defined in the conventional way as the RH profile weighted with the RH Jacobian. Cloud-contaminated measurements were determined and discarded equally as in the FIDUCEO CDR using the method suggested by^25^. Instead of selecting only pixels close to the nadir view of the satellite as for the FIDUCEO CDR, all pixels were used for the CM-SAF CDR. As discussed in the section on CDR processing this improves the sampling but results in a mixing of information about UTH from different altitude layers. For the CM-SAF CDR spatial averaging was performed in the same way as for the FIDUCEO CDR: UTH was separated into ascending and descending passes and then binned into 1° × 1° grid cells and averaged. For the comparison of the two CDRs, we calculate monthly averages of UTH from ascending and descending nodes and average these to get one combined monthly average.

When comparing UTH from the FIDUCEO CDR (UTH_FID_) and from the CM-SAF CDR (UTH_CMSAF_), we try to trace back the emerging differences to the above-named differences in the underlying FCDRs and in the processing of the two data records. For this purpose, we generated two additional versions of the FIDUCEO UTH CDR by changing aspects of the processing with respect to the original processing chain described in the section on CDR processing. For the first version, the UTH scaling parameters are changed to resemble the parameters used in the CM-SAF UTH CDR. Comparing the resultant UTH (UTH_CMSAFcoeff_) to UTH_FID_ reveals the effect of the different scaling parameters used for the FIDUCEO CDR (resulting from the new UTH definition). For the second version, the scaling parameters as well as the pixel selection of the CM-SAF CDR are imitated by including all pixels instead of using only near-nadir pixels. The UTH from this CDR version (UTH_CMSAFsampling_) is used to investigate the combined effect of the new scaling coefficients and the near-nadir only sampling we apply in the FIDUCEO CDR. Differences between the FIDUCEO CDR and the CM-SAF CDR that cannot be explained with the different scaling coefficients or the different sampling are a result of differences in the underlying FCDRs.

Figure 6a shows time series of monthly mean tropical mean UTH_FID_ for all satellite missions that are also included in the CM-SAF CDR: NOAA15, NOAA16, NOAA17, NOAA18, Metop-A and Metop-B. Time series of overlapping satellite missions agree within their uncertainties, which are indicated by the shaded areas around the monthly means. The time series of UTH_CMSAF_ is shown in Fig. 6b. Overall, there is good agreement between tropical mean UTH_FID_ and UTH_CMSAF_, confirming the validity of our method used to derive UTH. The difference plot in Fig. 7d shows that the absolute difference between tropical mean UTH_FID_ and UTH_CMSAF_ does not exceed 2% RH, except for the first months of the NOAA15 mission. For most satellite missions, UTH_FID_ tends to be about 1.6% RH lower than UTH_CMSAF_. Exceptions are NOAA15, for which UTH_FID_ is about 3% RH lower than UTH_CMSAF_ in the beginning of the mission and the difference is close to zero for the rest of the mission, as well as NOAA17, for which UTH_FID_ is about 0.8% RH lower than UTH_CMSAF_.

Inter-satellite biases differ in their magnitudes and signs between the FIDUCEO CDR and the CM-SAF CDR. For example, in the FIDUCEO CDR UTH from NOAA16 is systematically lower than UTH from NOAA17, whereas there is no bias between these two satellite missions in the CM-SAF CDR. In contrast, in our CDR UTH derived from NOAA17 and NOAA18 agree well, while UTH from NOAA17 is lower than UTH from NOAA18 in the CM-SAF CDR.

The difference between UTH_FID_ and UTH_CMSAFcoeff_ (Fig. 7b) is approximately 1.6% RH over all satellite missions, indicating that the use of our new UTH scaling coefficients offsets the monthly mean UTH by a positive, approximately constant value. The offset is slightly larger in months with high UTH than in months with low UTH, but these variations are small (smaller than 0.2% RH) compared to the mean offset.

The comparison of UTH_FID_ and UTH_CMSAFsampling_ (Fig. 7c) shows that near-nadir-only sampling affects the individual satellite missions in different ways, which can in part explain the differing inter-satellite biases in our CDR with respect to the CM-SAF CDR. For most of the missions the difference between UTH_FID_ and UTH_CMSAFsampling_ fluctuates around a mean negative value, indicating that the selection of only near-nadir pixels leads to slightly lower UTH values. This is consistent with the climatological C-shape of the tropical RH profile^36^, which implies slightly higher RH for off-nadir pixels that sample a slightly higher altitude.

More surprisingly, the mean difference between UTH_FID_ and UTH_CMSAFsampling_ is not uniform among satellite missions, particularly among the NOAA missions. Thus, inter-satellite biases change with a different selection of scan positions and therefore have to be scan-dependent. For NOAA15 and NOAA16 these scan-dependent biases additionally seem to be time dependent. When only near-nadir pixels are selected, for NOAA15 and NOAA17 the decrease in UTH is smaller than for the later instruments including NOAA18, Metop-A and Metop-B. In contrast, for NOAA16 the decrease in UTH is stronger and increases over time. Combined, the weaker decrease of NOAA17 UTH and the stronger decrease of NOAA16 UTH due to the near-nadir pixel selection result in a larger bias between NOAA16 and NOAA17 in UTH_FID_ than in UTH_CMSAF_. The instruments onboard NOAA15, NOAA16 and NOAA17 are known to suffer from radio frequency interference (RFI) from transmitters on-board the satellite^37–39^. This can explain the emerging scan- and time-dependent biases, since the effect of RFI is both scan- and time-dependent. An RFI correction was applied in the FIDUCEO FCDR to all three instruments^39^, which was shown to improve the consistency between instruments. However, for NOAA15 and NOAA17 only an early version of the correction scheme could be applied due to the lack of a reference month that is not affected by RFI. Moreover, the effect of the RFI correction has only been tested on the basis of *T*_*b*_ averaged over all scan positions. Thus, it is likely that scan- and time-dependent biases due to RFI still exist.

Figure 8 shows the geographical distribution of the differences between UTH_FID_ and UTH_CMSAF_. Differences are not distributed uniformly over the tropics. The amplitude of their spatial variations is on the order of 2% RH and hence of similar magnitude as the tropical mean differences (Fig. 7d). A main reason for the spatial variations in the differences lies in the different sampling applied in the production of the FIDUCEO CDR. This is confirmed by the fact that the same spatial patterns are visible in the difference between UTH_FID_ and UTH_CMSAFsampling_ (not shown). The near-nadir-only sampling of FIDUCEO results in a much lower number of observations in each grid cell and rarely occurring extreme events in certain regions that are captured by the CM-SAF CDR might be missing in the FIDUCEO CDR. Due to the non-linear relation between *T*_*b*_ and UTH this can translate into biases between FIDUCEO UTH and CM-SAF UTH.

There is a tendency of biases to be more negative in regions of climatologically high UTH, like the deep convective regions in the Inter Tropical Convergence Zone (ITCZ) and less negative in regions of low UTH, like the subtropical subsidence regions. This is reflected in a weak seasonal dependence of the tropical mean difference between UTH_FID_ and UTH_CMSAF_, which is apparent in Fig. 7d. Differences are more negative in months with a high average UTH and vice versa. The amplitude of these seasonal fluctuations in the difference is about 0.2% RH and hence small compared to the average difference.

To put the biases between UTH_FID_ and UTH_CMSAF_ into perspective, we can compare them to the state-of-the-art accuracy of space-borne UTH observations. It is impossible to define this accuracy precisely. However, we can take the difference between UTH derived from microwave and infrared sensors as an estimate. For the infrared instrument AIRS and the microwave instrument AMSU-B^40^ find a mean bias of about 3% RH between 60° S and 60° N (their Table 1) and regional biases on the order of ±10% RH (their Fig. 1). The biases between UTH_FID_ and UTH_CMSAF_ are about half as large. However, for two products from the same sensor type biases should of course be smaller than between products from different sensor types. Therefore, we overall judge the agreement between UTH_FID_ and UTH_CMSAF_ to be in line with expectations.

Our analysis has shown that part of the differences between UTH_FID_ and UTH_CMSAF_ can be explained by the differences in the CDR processing (scaling coefficients and selection of pixels). However, there are additional differences in the inter-satellite biases (e.g. larger biases between NOAA15 and NOAA16 as well as between NOAA17 and NOAA18 in the CM-SAF CDR) originating from differences in the underlying FCDRs. In summary, the underlying FCDRs and the CDR processing each explain about half of the total difference between UTH_FID_ and UTH_CMSAF_.

### Limitations: sources of uncertainty not included in the CDR

A major accomplishment of the FIDUCEO UTH CDR is the fact that it provides estimates of measurement uncertainty for all contained quantities. However, additional uncertainties arise with the level 2 and level 3 processing. They are not included in the CDR since a full understanding and quantification of each uncertainty requires thorough investigations, which should be part of future work. This section provides an overview of these additional uncertainties as well as rough estimates of their magnitude and recommendations for the CDR analysis. A more detailed discussion can be found in the Product User Guide (PUG) provided along with the UTH CDR.

#### Additional uncertainties at level 2

Uncertainties arising at level 2 are associated with the exponential model used for the transformation of *T*_*b*_ to UTH (Eq. (1)), because there are deviations from this exponential relationship in the real atmosphere. As a rough estimate of the resulting uncertainty in UTH one can use the retrieval standard deviation *σ*_Δ*UTH*_, apparent as the spread around the identity line in Fig. 4. The overall retrieval standard deviation *σ*_Δ*UTH*_ is is 2.9% RH for UTH ≤ 80% RH. For a more accurate estimation one should take into account that the magnitude of *σ*_Δ*UTH*_ varies over the possible range of UTH values (Fig. 5) and the uncertainty estimate should hence depend on UTH itself. Note that using this uncertainty estimate for monthly grid cell averages of UTH (level 3 UTH) represents a simplified approach since several instantaneous UTH values with different uncertainties entered these averages.

#### Additional uncertainties at level 3

Uncertainties in the monthly averages of *T*_*b*_ and UTH arise because they are estimated from only a few satellite overpasses. A grid cell in the tropics is typically observed by about 12 to 14 satellite overpasses per month for the newer AMSU-B and MHS missions (Fig. 9). In the earlier SSMT-2 missions (F11, F12, F14, F15), however, large data gaps result in a poorer sampling. There are months with less than two average satellite overpasses per grid cell in all four missions. Hence, the time period before 1999, in which only SSMT-2 observations are available, should be used very cautiously. A related uncertainty results from the fact that a satellite always observes a given point on Earth at the same local time due to its sun-synchronous orbit and therefore always observes the same phase of the diurnal cycle. Thus, the monthly averages derived from these observations are only valid for a certain time of day. The resulting uncertainty in the monthly average depends on the amplitude of the diurnal cycle of UTH in the considered grid cell^41,42^. found diurnal amplitudes on the order of 1% RH over ocean and 4% RH over land regions using observations from geostationary satellites. These amplitudes can be taken as a rough estimate for the uncertainty due to the diurnal cycle. Estimating the uncertainty more precisely or even correcting for the diurnal cycle would require an exact knowledge of the temporal course of the diurnal cycle. In order to improve the sampling and to get the best estimate of the true monthly average, measurements from ascending and descending satellite overpasses as well as measurements from all available satellite missions with different equator-crossing times should always be combined.

Uncertainties in the long-term trend of *T*_*b*_ and UTH can result from a drift in the satellite orbit. With the exception of Metop-A and Metop-B, which are actively stabilised, all satellites included in the UTH CDR are subject to an orbit drift, which is reflected in a changing equator-crossing time over the course of the satellite mission. As a consequence, the observed phase of the diurnal cycle changes. Such an aliasing of the diurnal cycle can lead to artificial trends when long time scales are analysed. A comparison of the *T*_*b*_s measured by the stabilised satellite Metop-A and the drifting satellite NOAA18 (Fig. 6) suggests that such artificial trends are small when ascending and descending satellite overpasses are combined to one time series.

Another problem arising at level 3 is a dry bias (or “clear-sky” bias) in UTH^25,43^ resulting from the cloud filtering, which systematically removes moist pixels. To illustrate this bias in the FIDUCEO UTH CDR, the difference between monthly tropical mean *T*_*b*full_ and *T*_*b*filtered_ is shown in Fig. 10. For most missions *T*_*b*filtered_ is about 0.2 K warmer than *T*_*b*full_, corresponding to a fractional bias of about −2% in tropical mean UTH. This only represents an upper limit for the dry bias, because the cloud contaminated pixels included in *T*_*b*full_ appear colder (moister) than they actually are. More importantly, however, Fig. 10 reveals that for some instruments the difference between *T*_*b*full_ and *T*_*b*filtered_ is significantly larger than 0.2 K. Affected instruments are SSMT-2 on F14 (after 2001), AMSU-B on NOAA15 (whole mission), AMSU-B on NOAA16 (after 2006) and MHS on NOAA19 (whole mission). Those missions are affected by strong instrumental noise^44^, which leads to a broader distribution of measured *T*_*b*_s. For those instruments, cutting off *T*_*b*_s below a certain threshold during the cloud filtering leads to a larger change in mean *T*_*b*_ than for instruments with less noise. In order to avoid time dependent biases when a climatological time series is created, the affected time periods should be excluded.

A time series of tropical mean UTH created following the guidelines given above (i.e. combining measurements from ascending and descending overpasses and from overlapping satellite missions as well as excluding time periods affected by strong instrument noise) has shown good agreement with a UTH time series from HIRS measurements^45^ except from the first six years of the data record, where data coverage is poor.

## Data Availability

The code used for the processing of the FIDUCEO Microwave UTH CDR is available on GitHub (https://github.com/FIDUCEO/CDR_UTH).
